# High-throughput amplicon sequencing datasets of coastal sediments from three locations of the Gulf of Mexico, USA

**DOI:** 10.1016/j.dib.2023.108895

**Published:** 2023-01-11

**Authors:** George B.H. Green, Angelo DePaola, Jason G. Linville, Casey D. Morrow, Asim K. Bej

**Affiliations:** a Department of Biology, The University of Alabama at Birmingham, Birmingham, AL 35294, United States; b DePe Oysters, LLC, Coden, AL 36523, United States; c Department of Criminal Justice, The University of Alabama at Birmingham, Birmingham, AL 35294, United States; d Department of Cell, Developmental and Integrative Biology, The University of Alabama at Birmingham, Birmingham, AL 35294, United States

**Keywords:** Metagenome, QIIME2, Illumina, 16S rRNA gene, Oysters, Sediment, U.S. Gulf Coast

## Abstract

We present high-throughput amplicon sequence (HTS) datasets of the purified microbial metacommunity DNA of coastal surface sediments from Portersville Bay (PVB) (*n* = 3), Bayou La Batre (BLB) (*n* = 3), and Mobile Bay (MOB) (*n* = 3) of the U.S. Gulf of Mexico (U.S. Gulf Coast). The PVB samples were collected from the oyster aquaculture Shellevator™ system; the BLB samples were from locations on the shoreline adjacent to wild oysters attached to rocks and likely polluted from sewage and possibly chemical contamination from boats, shipyards, and seafood processing facilities; and MOB samples were adjacent to aquaculture oysters in bottom cages. The amplicons of the V4 hypervariable segment of the 16S rRNA gene from each sample were sequenced on an Illumina MiSeq to generate these HTS datasets. The raw sequences were quality-checked, demultiplexed into FASTQ files, denoised using DADA2, and subsampled. Then, the FASTA formatted sequences were assigned the taxonomic ids to amplicon sequence variants (ASVs) against the silva-138–99-nb-classifier using the Quantitative Insights Into Microbial Ecology (QIIME2 v2022.2). The applicability of the HTS datasets was confirmed by microbial taxa analysis at the phylum level using the "qiime taxa collapse" command. All HTS datasets are available through the BioSample Submission Portal under the BioProject ID PRJNA876773 (https://www.ncbi.nlm.nih.gov/bioproject/?term=PRJNA876773).

**Specification Table**

**Table:** 

Subject	Biological Sciences
Specific subject area	Microbiology: Microbiome. High-throughput amplicon metagenome sequence datasets of the 16S rRNA gene (V4 hypervariable segment) from three locations of the coastal surface sediments of the U.S. Gulf Coast.
Type of data	Figures and Tables
How the data were acquired	Illumina MiSeq platform with 250 paired-end kits.
Data format	Raw HTS FASTQ Format.
Description of data collection	The samples from diverse estuarine surface sediments of the U.S. Alabama Gulf Coast represent the (1) oyster aquaculture Shellevator™ systems (Portersville Bay (PVB); 30°22′16″N 88°13′52″W); (2) shoreline adjacent to wild oysters attached to rocks and likely polluted from sewage and possibly chemical contamination from boats, shipyards, and seafood processing facilities (Bayou La Batre (BLB); 30°22′58.2″N 88°16′13.3″W); and (3) adjacent to the aquaculture oysters in bottom cages (Mobile Bay (MOB); 30°25′32″N 88°6′14″W).
Data source location	UAB Department of Criminal Justice, 1201 University Blvd., UBOB, Birmingham, AL 35294, USA (33°25′57″N, 86°48′36″W). Microbial metacommunity DNA was prepared and sequenced at the UAB Department of Genetics, Heflin Center Genomics Core, School of Medicine, the University of Alabama at Birmingham, 705 South 20th Street, Birmingham, AL 35294, USA. (33°21′40″N, 87°53′91″ W).
Data accessibility	Raw data [1] corresponding to the nine HTS sequences from three sediment sample locations, *Portersville Bay (PVB) (n* = 3), *Bayou La Batre (BLB) (n* = 3), and *Mobile Bay (MOB) (n* = 3) of the U.S. Gulf Coast are available at the NCBI's Bioproject database (https://www.ncbi.nlm.nih.gov/bioproject/) under the *BioProject ID PRJNA876773 (*https://www.ncbi.nlm.nih.gov/bioproject/?term=PRJNA876773*).*

## Values of the Data

• The HTS datasets will help expand our knowledge about the diversity of microbial communities in sediments adjacent to wild and aquaculture oysters along the U.S. Gulf Coast.
• The metagenome datasets exhibited diverse microbial communities and relative abundances, including some that were relatively unique to the sediment samples from each location, thus confirming the applicability of the data.
• The raw HTS metagenome datasets shared through the publicly available sequence repository (NCBI) will allow researchers to analyze the influence of oysters and oyster aquaculture on microbial communities of adjacent sediments in ecosystems often impacted by various pollution sources.
• The raw HTS metagenome datasets can be used by broad scientific communities for their own research goals, including microbial and environmental forensics such as tracing the source of oil spills, toxic chemicals, and pathogens affecting human health, coastal communities, and the ecosystem.

## 1 Objective

The objective of this work was to report the V4 hypervariable segment of the16S rRNA gene amplicon metagenome sequence datasets from coastal sediment samples adjacent to wild and aquaculture oysters from three diverse ecologically and commercially important locations on the U.S. Gulf Coast. The raw HTS datasets have been deposited to the NCBI BioProject portal under the ID PRJNA876773 and are available for public use. The quality and applicability of these HTS datasets determined through the bioinformatics tools presented in this report will benefit broad scientific communities for their future research goals.

## 2 Data Description

**Fig. 1:**
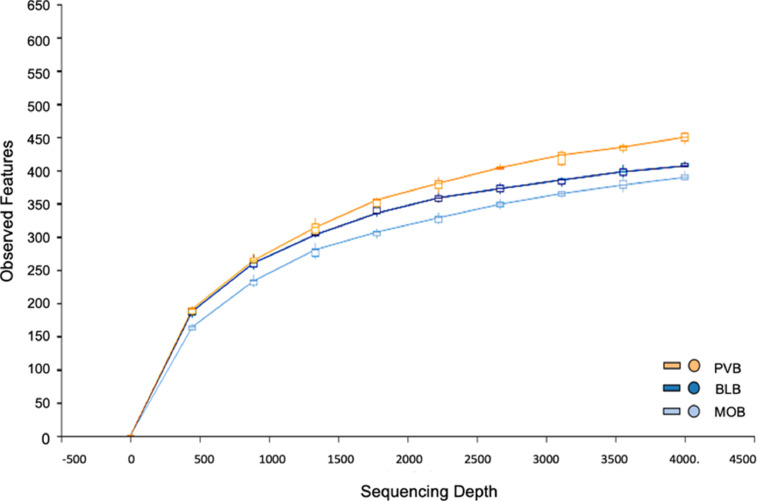
Rarefaction curve analysis of the high-throughput sequence (HTS) datasets of the three U.S. Gulf Coast sediment samples displaying the observed features (y-axis) plotted against the number of sequences (Sequence Depth) (x-axis) per sample. Observed features were generated and plotted using the QIIME2 (v2022.2) view command. Each line represents the median values from three replicates of each sample. Sample designations are as follows: PVB = Portersville Bay, AL, USA (30°22′16″N 88°13′52″W); BLB = Bayou La Batre, Dauphin Island, AL, USA (30°22′58.2″N 88°16′13.3″W); and MOB = Mobile Bay, Coden, AL, USA (30°25′32″N 88°6′14″W).

The HTS datasets (NCBI Bioproject ID PRJNA876773) represent the microbial metagenomics of sediments (top 1 cm) in proximity (<5 cm) to oysters (*Crassostrea virginica*) in three coastal Alabama sites on the U.S. Gulf Coast. Metagenome data was generated from sediment samples (1) under aquaculture oysters on the deck of the Shellevator™ system in National Shellfish Sanitation Program (NSSP) conditionally approved area located at Portersville Bay, AL (30°22′16″N 88°13′52″W) (*n* = 3); (2) next to wild oysters attached to rocks on shore in NSSP prohibited area at Lightning Point at the outflow of the Bayou La Batre, AL (30°22′58.2″N 88°16′13.3″W) (*n* = 3) likely polluted from sewage, chemical contamination from boats, shipyards, and seafood processing facilities; and (3) next to bottom cages of cultured oysters in NSSP conditionally approved area along the western shore of Mobile Bay, Coden, AL (30°25′32″ N 88°6′14″W) (*n* = 3). We used amplicon sequencing of the V4 hypervariable segment of the 16S rRNA gene on an Illumina MiSeq platform to generate the metagenome data [2]. The statistics of the HTS data are presented in Table 1. The rarefaction analysis of the HTS data constructed at 3% sequence variation from each sample reached saturation, thus confirming the quality and usefulness of these datasets (Fig. 1).

**Table 1:** The high-throughput sequence statistics were generated by QIIME2 (v2022.2) tools. The table lists the total number of raw sequence reads followed by filtered, denoised, merged, and non-chimeric sequence data. Sample designations are as follows: PVB = Portersville Bay, AL, USA (30°22′16″N 88°13′52″W); BLB = Bayou La Batre, Dauphin Island, AL, USA (30°22′58.2″N 88°16′13.3″W); and MOB = Mobile Bay, Coden, AL, USA (30°25′32″ N 88°6′14″W). The numbers after each sample designation represent the replicates (*n* = 3 for each location).

Sample	Raw Sequence Reads	Filtered Sequences	Denoised Sequences	Merged Sequences	Non-Chimeric Sequences
PVB-1	118,149	55,405	52,149	47,088	46,586
PVB-2	130,927	54,053	51,452	42,847	39,362
PVB-3	100,913	46,892	43,121	38,634	37,891
BLB-1	91,166	31,309	28,457	22,407	22,191
BLB-2	96,074	43,375	39,842	32,717	32,436
BLB-3	117,154	30,006	27,007	21,871	21,269
MOB-1	115,776	43,051	38,193	30,428	29,887
MOB-2	134,137	57,553	51,659	42,304	41,911
MOB-3	105,316	37,456	34,933	29,279	29,174
**Total**	**1009,612**	**399,100**	**366,813**	**307,575**	**300,707**

**Fig. 2:**
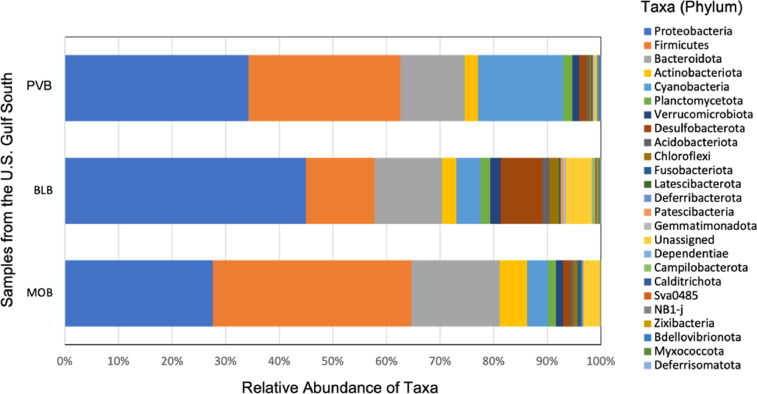
Relative abundance distribution of top 25 taxa at the phylum level of the sediment samples (*n* = 3 for each sample) from the U.S. Gulf Coast. The taxonomic identities were assigned based on the amplicon sequencing of the V4 hypervariable segment of the 16S rRNA gene through the SILVA v138 (silva-138–99-nb-classifier.qza) database. The analysis was performed using the Quantitative Insights into Microbial Ecology (QIIME2, v2022.2) tools and graphed using Microsoft Excel software (Microsoft Corporation, Redmond, WA, USA). The color codes in the stacked column bar correspond to the color of the phylum designation on the right panel. Sample designations are as follows: PVB = Portersville Bay, AL, USA (30°22′16″N 88°13′52″W); BLB = Bayou La Batre, Dauphin Island, AL, USA (30°22′58.2″N 88°16′13.3″W); and MOB = Mobile Bay, Coden, AL, USA (30°25′32″ N 88°6′14″W).

The metagenomic data at the phylum level revealed the highest relative abundance of Proteobacteria in BLB samples (45%) as compared to PVB (34%) and MOB (28%) (Fig. 2; Table 2). In contrast, Firmicutes were highest in MOB samples (37%), followed by PVB samples (28%) and then BLB samples (13%). The Bacteroidota represented a slightly higher abundance in MOB samples (16%) compared to BLB (13%) and PVB (12%). Cyanobacteria were 3–4 times more abundant in PVB samples (16%) than in other sites. Desulfobacterota in the BLB samples (8%) was 8-fold higher than in other sites. The rest of the phyla distribution varied at or below 5%. Overall, the distribution of the top 25 phyla and the relative abundances across all three samples presented in Fig. 2 and Table 2 confirm the quality and the applicability of the datasets presented in this manuscript.

**Table 2:** Statistical analysis of the representative high-throughput sequences from the U.S. Gulf Coast sediment samples aligned to SILVA v138 taxonomic database (silva-138–99-nb-classifier.qza). Taxonomic assignments were performed using QIIME2 (v2022.2), and percentages were calculated across all samples (*n* = 3 for each sample). The top 25 phyla were selected based on total relative abundance. The sample designations are as follows: PVB= Portersville Bay, AL, USA (30°22′16″N 88°13′52″W); BLB = Bayou La Batre, Dauphin Island, AL, USA (30°22′58.2″N 88°16′13.3″W); and MOB = Mobile Bay, Coden, AL, USA (30°25′32″ N 88°6′14″W).

Phylum	PVB	BLB	MOB
Proteobacteria	34.29%	45.01%	27.63%
Firmicutes	28.28%	12.78%	36.99%
Bacteroidota	12.03%	12.55%	16.49%
Actinobacteriota	2.49%	2.69%	5.10%
Cyanobacteria	15.83%	4.52%	3.92%
Planctomycetota	1.74%	1.78%	1.49%
Verrucomicrobiota	1.29%	1.97%	1.33%
Desulfobacterota	1.44%	7.86%	1.43%
Acidobacteriota	0.46%	1.33%	0.56%
Chloroflexi	0.35%	1.65%	0.67%
Fusobacteriota	0.05%	0.08%	0.66%
Latescibacterota	0.21%	0.29%	0.17%
Deferribacterota	0.01%	0.03%	0.14%
Patescibacteria	0.08%	0.47%	0.18%
Gemmatimonadota	0.28%	0.52%	0.09%
Unassigned	0.31%	4.76%	2.94%
Dependentiae	0.06%	0.04%	0.02%
Campilobacterota	0.20%	0.55%	0.07%
Calditrichota	0.03%	0.08%	0.02%
Sva0485	0.09%	0.27%	0.01%
NB1-j	0.16%	0.16%	0.05%
Zixibacteria	0.00%	0.11%	0.00%
Bdellovibrionota	0.25%	0.08%	0.01%
Myxococcota	0.05%	0.32%	0.01%
Deferrisomatota	0.03%	0.11%	0.00%

## 3 Experimental Design, Materials and Methods

### 3.1 Sample Collection

The surface sediment samples (∼15 g each) from PVB, BLB, and MOB were collected from the intertidal zones in coastal Alabama of the U.S. Gulf Coast during low tide between 12:30–2:30 pm on November 8, 2021. The samples were collected in sterile 50 ml Corning™ polyproline Falcon™ tubes (Corning™ 352,070; Fisher Scientific, Waltham, MA), stored cold at 4 °C in 10 ml 70% (v/v) ethyl alcohol, and transported to UAB for metagenomic studies.

The sediment samples from the PVB (*n* = 3) (Portersville Bay, AL; Salinity: 23.7 parts per thousand (ppt); Temperature: 22.5 °C; GPS: 30°22′16″N 88°13′52″W) were collected ∼5 cm under aquaculture oysters on fiberglass deck of Shellevator™ system (https://www.shellevator.com). The Portersville Bay site is ∼100 m from the northern shore of the Mississippi Sound ∼10 Km west of Mobile Bay and less subject to salinity fluctuations and other consequences of rainfall runoff from the Alabama watershed. It is impacted by pollution from local rainfall runoff and rainfall-related discharge from the BLB [3,4]. PVB salinity runs 5–10 ppt, greater than the MOB site, and ranges from 5 to 25 ppt. PVB is surrounded mainly by marsh shores and supports healthy populations of finfish and crabs [5]. Although abundant wild oyster production on the bottom has ended in the past few decades, PVB still supports several off-bottom oyster farms [6].

The samples collected from BLB (*n* = 3) (Bayou La Batre, Lightning Point, AL; Salinity: 19.8 ppt; Temperature: 23.9 °C; GPS: 30°22′58.2″N 88°16′13.3″W) likely had pollution from sewage, possibly chemical contamination from boats, shipyards, and seafood processing facilities [4]. The BLB site borders the Mississippi Sound ∼5 Km west of the PVB site and has a similar salinity profile. The sediment sample was collected 1–2 m from shore in a cluster of oysters attached to rocks near the mouth of the Bayou downstream from numerous large seafood processing plants and shipyards and within 1 Km of the Sewage Treatment Plant (STP) effluent discharge pipe [3].

The third set of samples was collected adjacent to aquaculture oysters in the bottom cage at the farm in the MOB (*n* = 3) (Mobile Bay; Salinity: 9.8 ppt; Temperature: 22.7 °C; GPS: 30°25′32″N 88°6′14″W). The Mobile Bay site (1–2 m from shore) is heavily influenced by rainfall runoff from its watershed that covers nearly the entire state of Alabama, which is the third largest in the lower 48 states. Flooding during the winter/spring rainy season and periodic tropical storms reduces salinity and introduces eroded soils laden with fertilizer and microorganisms, including pathogens [7,8]. Suspended sediments reduce visibility, and high nutrient levels stimulate algal blooms and exacerbate hypoxia [9]. Over the past several decades, abundant submerged aquatic vegetation has disappeared, and commercially important benthic fauna, including oysters, crabs, and brown shrimp, no longer support commercial harvest in much of the Bay [10], [11], [12], [13]. Over this period, most of the shore has been hardened with sea walls with little remaining marshes [14]. The salinity and the temperature were recorded from all locations using a YSI Pro30 Conductivity meter (YSI Incorporated, Yellow Springs, Ohio; https://www.ysi.com/pro30) for all samples.

### 3.2 High-Throughput Sequencing

The metacommunity DNA from the sediment samples was purified using the Zymo Research kit (Irvine, CA, USA) per the manufacturer's instructions. Purified DNA was quantified, and purity was determined using a NanoDrop (Nanodrop One C, Thermo Fisher Scientific, Madison, WI, USA). The purified DNA was then subjected to HTS using Illumina MiSeq and 250 bp paired-end kits (Illumina, Inc., San Diego, CA, USA). We targeted the V4 hypervariable segment of the 16S rRNA gene for the HTS [2]. The purification, quantity, and quality assessments of the purified sediment metacommunity DNA and HTS were performed at the UAB Microbiome Resource Center (https://www.uab.edu/medicine/microbiome/resources/cores) and Heflin Center for Genomic Sciences (https://www.uab.edu/hcgs/). The demultiplexed, FASTQ formatted sequences were then deposited to the National Center for Biotechnology Information (NCBI) Sequence Read Archive (SRA) under Bioproject ID PRJNA876773 (https://www.ncbi.nlm.nih.gov/bioproject/?term=PRJNA876773) assigned to samples from PVB, BLB, and MOB.

### 3.3 Taxonomic Classification

The HTS datasets were demultiplexed into FASTQ files and then imported into Quantitative Insights Into Microbial Ecology (QIIME2; v2022.2) using the cassava 1.8 paired-end demultiplexed fastq format [15]. The raw sequence reads were quality-checked using the ``qiime demux summarize'' function and denoised using DADA2 (q2-dada2 denoise-paired) [16]. The ``q2- feature-table tabulate-seqs'' command was used to generate representative sequences (rep-seqs). The multiple sequence alignment program (MAFFT ver.6) aligned the amplicon sequence variants (ASVs) [17], and the sequence output was piped into the Approximate-Maximum-Likelihood Trees (fasttree2 - q2-phylogeny) [18]. The Alpha and Beta diversity statistics were determined by the ``core-metrics-phylogenetic'' command [19]. The samples were rarefied to a minimum of 41,356 sequences per sample (subsampled without replacement). Taxonomic identifications (ids) represented the respective sequences assigned to ASVs against the Silva-138–99-nb-classifier [20]. The taxonomy generated was collapsed (Levels 1–7) using the ``qiime taxa collapse'' command [15] and presented here at the phylum level (Fig. 2; Table 2) to confirm the applicability of the HTS datasets for the microbiome studies.

## Ethics Statements

The data presented in this study did not involve using human or animal subjects or social media platforms. Therefore, no ethical statements as per the journal policy were required for the data.

## Data Availability

The metagenome datasets corresponding to the nine HTS sequences from three sediment sample locations are publicly available at the NCBI's Bioproject database (https://www.ncbi.nlm.nih.gov/bioproject/) under the BioProject ID PRJNA876773 (https://www.ncbi.nlm.nih.gov/bioproject/?term=PRJNA876773).

## CRediT authorship contribution statement

**George B.H. Green:** Formal analysis, Data curation, Writing – original draft. **Angelo DePaola:** Resources, Formal analysis, Writing – review & editing. **Jason G. Linville:** Writing – review & editing. **Casey D. Morrow:** Funding acquisition, Resources, Writing – review & editing. **Asim K. Bej:** Conceptualization, Formal analysis, Data curation, Supervision, Writing – review & editing.

## Declaration of Competing Interest

The authors declare no known competing financial interests or personal relationships that could have appeared to influence the work reported in this paper.
